# Research on the social capital, knowledge quality and product innovation performance of knowledge-intensive firms in China

**DOI:** 10.3389/fpsyg.2022.946062

**Published:** 2022-10-06

**Authors:** Xia Zhou, Min Min, Zhen Zhang

**Affiliations:** ^1^School of Business Administration, South China University of Technology, Guangzhou, China; ^2^Business School, Shandong Normal University, Jinan, China

**Keywords:** knowledge quality, product innovation performance, structural social capital, relational social capital, cognitive social capital

## Abstract

Given the importance of social capital for the success of knowledge-intensive firms (KIFs), scholars have investigated how social capital promotes product innovation through knowledge transfer. However, in contrast to the quantity of transferred knowledge, the role of knowledge quality has been largely ignored. Drawing on the knowledge-based view (KBV) of the firm, this study explores the influences of structural, relational, and cognitive social capital on product innovation and the mediating role of knowledge quality. A questionnaire-based survey was conducted from firm executives and core members of R&D department and data of 159 Chinese KIFs were obtained. Partial least squares-structural equation modeling was used for hypotheses testing. The results reveal that relational capital and cognitive capital have positive effects on product innovation performance, which are mediated by knowledge quality. However, the effect of structural social capital on knowledge quality is not significant. The results contribute to literature on firm innovation by examining how social capital indirectly affects product innovation performance through the quality of transferred knowledge. Moreover, the conclusions can help top management of KIFs to design more effective informal knowledge management approaches according to differentiated effects of three types of social capital on knowledge quality.

## Introduction

Globalization and technological progress have provided a series of opportunities and challenges for firms, and an increasing number of firms have realized the importance of product innovation in the context of improving performance and competitive advantages (Najafi-Tavani et al., 2018; Mu et al., 2021). The innovation process of firms depends on their ability to collect and use resources from multiple sources, both external and internal (O’Cass and Sok, 2014; La Rocca et al., 2016; Khan et al., 2020; Salas-Vallina et al., 2020; Mazzucchelli et al., 2021; Fayad and El Ebrashi, 2022). Therefore, some researchers have encouraged firms to construct social ties and strengthen social capital with other firms in social networks (Motohashi, 2005; Molina-Morales and Martínez-Fernández, 2010). Because they believe that social capital, which is consisted of a set of resource-embedded relationships among social actors, is conducive to the exchange of external resources and the expansion of the existing knowledge bases (Engelman et al., 2017; Najafi-Tavani et al., 2018; Presutti et al., 2022). If firms, especially KIFs, have more social capital, they will be likely to obtain more resources, develop market-oriented products and services, and become more competitive (Carmona-Lavado et al., 2010; Eiteneyer et al., 2019; Ganguly et al., 2019; Cappiello et al., 2020; Duodu and Rowlinson, 2020).

Social capital includes three dimensions, namely, interactions and social relationships (structural social capital), relationships of mutual respect and trust (relational social capital), and shared values and goals of network members (cognitive social capital) (Nahapiet and Ghoshal, 1998). Each dimension may have a specific impact on innovation (Ortiz et al., 2018; Mazzucchelli et al., 2021). However, most previous studies have regarded social capital as a whole to investigate its impact on product innovation. Therefore, this study aims to explain the relationship between social capital and product innovation performance more completely by examining the specific and independent effects of the three dimensions of social capital on product innovation performance.

We further use the knowledge-based view (KBV) as the basis for our research to understand the role of knowledge transfer in the relationship between social capital and product innovation. The KBV holds that a firm is a set of mechanisms that improves knowledge processes (e.g., inter-firm coordination), and the core competences of a firm are enhanced through successive knowledge processes (knowledge transfer, integration and creation) (Blome et al., 2014). Although knowledge is crucial to the development of firms (Boeker et al., 2021), our understanding of the role of inter-firm knowledge process in the relationship between firm’s social capital and product innovation performance is not insufficient. Researchers have recognized that knowledge transfer has a multidimensional nature and, that it includes knowledge quantity and quality (Kyoon Yoo, 2014; Liu et al., 2017). However, previous research on the relationship between social capital and product innovation has mainly focused on the mediating role played by the quantity of knowledge transferred (Filieri et al., 2014); and few studies on the knowledge quality (Boeker et al., 2021; Moser and Deichmann, 2021). Actually, the current studies indicate that the quantity of knowledge transfer does not always improve innovation performance (Belenzon and Berkovitz, 2010). Knowledge quality reflects the suitability of knowledge to a task at hand (March, 1991). Only when knowledge is applicable to the current innovation task can it promote the improvement of innovation performance (Corral de Zubielqui et al., 2019). Therefore, when firms use external knowledge resources for product innovation, they should pay more attention to whether the knowledge acquired through inter-firm knowledge transfer is valuable for product innovation (Kyoon Yoo, 2014; Han et al., 2018). The above discussion leads to the two research questions (RQs) of this paper:

RQ1: In China, can firm social capital improve product innovation performance?

RQ2: In China, does knowledge quality mediate the relationship between social capital and product innovation?

The goal of this paper is to contribute to the research on the social capital and innovation of firms. First, to address the contextual characteristics of social capital, we collected survey data from Chinese knowledge-intensive firms to examine the relationship between firm social capital and product innovation in the Chinese context. Second, based on the KBV, we examined the mediating role of knowledge quality in the relationship between social capital and product innovation, providing insights for further research on the internal mechanism of firm’s social capital on innovation.

## Theoretical background and research hypotheses

### Social capital, knowledge quality and product innovation performance

Given that knowledge has been regarded as a key driver of a firms’ long-term advantage (Leal-Rodríguez et al., 2014), this paper adopts the KBV of the firm as a theoretical anchor to understand the relationship between social capital and product innovation performance, as well as the underlying mechanisms of this relationship. The KBV treats firms or organizations as mechanisms to improve firms’ knowledge processes (Un and Asakawa, 2015) and provides a theoretical basis to interpret the salient roles that organizational knowledge and governance mechanisms play in firm innovation (Grant, 1996a). Firms pursuing product innovation provide formal and informal governance mechanisms to motivate units both inside and outside the firm to transfer novel knowledge (Zhang and Min, 2019). For example, to realize knowledge transfer across organizational boundaries, firms use social capital as an effective informal governance mechanism to collaborate with external firms (Zhang and Min, 2021). In addition, knowledge quality is also an important dimension of knowledge transfer. In contrast to the knowledge quantity, knowledge quality is rarely paid attention to. Building on the viewpoints of the KBV, this paper untangles the linkage among social capital, knowledge quality and product innovation.

“Product innovation” refers to the new product development process, including technical design, R&D, manufacturing and management (Danneels, 2002). In this study, we adopt Alegre et al. (2006) research and hold that product innovation performance includes two dimensions: effectiveness and efficiency. Innovation effectiveness reflects the degree to which an innovation is successful, and innovation efficiency reflects the efforts carried out to achieve that degree of success. Currently, many product innovation studies use these two widely validated dimensions.

“Social capital” refers to certain features of social organizations, such as their networks, norms and trust that are conducive to coordination, cooperation and mutual benefit. Nahapiet and Ghoshal (1998) pointed out that social capital is embedded within mutually recognized networks. Through connections or others provided by the network, organizations or individuals can access external resources, such as information, opportunities, social status and reputation. Therefore, social capital includes not only networks but also the resources that can be accessed through these networks. Nahapiet and Ghoshal (1998) further proposed that there are three dimensions of social capital, namely, the structural dimension, relational dimension and cognitive dimension, which have been widely applied. This study attempts to consider the fundamental mechanism underlying the relationships between all three dimensions of social capital and product innovation performance. The structural dimension refers to the presence or absence of network ties or network configurations between actors and describes linkage patterns in terms of their density, connectivity and hierarchy (Nahapiet and Ghoshal, 1998). The relational dimension describes the assets embedded in social relationships, focusing on special relationships that affect the behavior of actors such as those involving trust and trustworthiness (Tsai and Ghoshal, 1998). The cognitive dimension is described as a shared value system (including common language coding, shared goals and common understandings of things) that facilitates interactions between actors in special social contexts (Nahapiet and Ghoshal, 1998; Ganguly et al., 2019).

“Knowledge quality” refers to the applicability of knowledge (Kyoon Yoo, 2014). Waheed and Kaur (2016) identified some important characteristics of knowledge quality, including adaptability, innovativeness, applicability, expandability, justifiability and authenticity. Knowledge management research has begun to pay attention to knowledge quality (Haas and Hansen, 2007; Kyoon Yoo, 2014; Corral de Zubielqui et al., 2019), and has argued that the success of knowledge transfer may depend on recipients’ satisfaction with the transferred knowledge (Ghobadi and D’Ambra, 2012). This study defines knowledge quality as the firm’s satisfaction with the transferred knowledge and how useful it is in the product innovation process.

### Social capital and product innovation performance

The foundation of the KBV is its emphasis that knowledge is the main source of value (Grant, 1996b; Blome et al., 2014). Innovation requires firms to integrate various types of knowledge (Grant, 1996a; Landry et al., 2002). The KBV indicates that the knowledge required for product innovation is widely distributed inside and outside a firm (Blome et al., 2014; Najafi-Tavani et al., 2018). To integrate this knowledge, it is necessary for firms to cope with organizational boundaries and interact with diverse organizations through social network (Landry et al., 2002; Grant and Baden-Fuller, 2004).

Current studies show that the structural dimension of social capital (contact frequency and interaction type) has an impact on firms’ willingness to transfer and integrate external resource (Yli-Renko et al., 2001; Inkpen and Tsang, 2005; Zhou et al., 2014; Ortiz et al., 2018). A firm with a strong inter-organizational connection enjoys a higher status and more power in its social network, faster resource flows and have more opportunities to obtain valuable resources (Tsai, 2006; Presutti et al., 2016; Gerke et al., 2021). The capabilities or resources acquired through frequent interactions are critical to improving new product quality and shortening the time to market (Pérez-Luño et al., 2011).

The relational dimension of social capital refers to the quality of the interactions derived from the structural dimension (Carmona-Lavado et al., 2010), and its key characteristics are trust and trustworthiness (Ganguly et al., 2019). Trust plays a vital role in the process of interaction (Mazzucchelli et al., 2021). Establishing high levels of credibility in social networks can encourage firms’ partners to give them maximum resource commitments (Pérez-Luño et al., 2011). A high level of trust among participants positively influences the strength and efficiency of information exchange and increases the quality of social interactions, thus creating an environment conducive to innovation (Lane et al., 2001). Therefore, product innovation performance is likely to improve as relational social capital increases.

The cognitive dimension, understanding and exchange of knowledge need a common cognitive reference (Kang et al., 2007). Therefore, in social networks, the cognitive dimension of social capital is of great significance for knowledge identification and acquisition because it allows partners (individuals or organizations) to understand each other through common goals and language (Ortiz et al., 2018). Shared interests and visions can foster the willingness of organizations or individuals to exchange resources that can be used to support them to jointly create innovative solutions (Chow and Chan, 2008; Mazzucchelli et al., 2021).

Thus, we propose the following:

H1a: Structural social capital has a positive effect on product innovation performance in Chinese knowledge-intensive firms.

H1b: Relational social capital has a positive effect on product innovation performance in Chinese knowledge-intensive firms.

H1c: Cognitive social capital has a positive effect on product innovation performance in Chinese knowledge-intensive firms.

### The mediating role of knowledge quality

Knowledge transfer has been recognized as a key means by which firm competitive advantage can be shaped based on innovation (Ortiz et al., 2018). Highly specialized, timely and accurate knowledge is the key factor for firms to gain competitive advantages in a dynamic and innovative environment (Di Vaio et al., 2021). However, the continuous acquisition of such knowledge through internal development of a firm often faces many difficulties (García-Sánchez et al., 2017; Goyal et al., 2020). Inter-organizational knowledge transfer is a platform for firms to acquire external knowledge, but it is easy to produce distorted information or knowledge (Nazam et al., 2020). Therefore, attention needs to be paid to the quality of transferred knowledge (Bloodgood, 2019). The sources and types of external knowledge are diverse. It has been documented that the quality of external knowledge acquired by a firm is complementary to the resources and capabilities that it owns (Laursen et al., 2012; García-Sánchez et al., 2017; Ortiz et al., 2018). Transfers of knowledge that is highly specialized and adaptive must be carried out in closely interactive environments (Maula et al., 2003). The strong social network resources obtained by firms through frequent interaction with external organizations can help to generate more valuable knowledge flow (Steinberg et al., 2017; Ortiz et al., 2018).

Many studies have examined the relationship between knowledge transfer and trust. For example, Massaro et al. (2019) found that in the case of networks of SME networks, trust leads to a higher level of knowledge transfer between firms. High-level communication between firms is possible due to trust, which is particularly important in the case of high-value knowledge transfer (Diallo and Thuillier, 2005). The higher the degree of expertise need to acquire knowledge is, the greater the demand for interactions between firms and the higher the level of trust required for knowledge transfer (Auetsch et al., 2011). In addition, trust minimizes opportunistic motivation and makes enables actors to exchange more valuable knowledge (Nahapiet and Ghoshal, 1998). Therefore, the high level of relational social capital owned by firms in social networks may be conducive to the acquisition of high-quality knowledge.

In social networks, the acquisition of external resources requires firms to inform and understand the knowledge of other organizations (Expósito-Langa et al., 2015). Shared goals and visions facilitate faster communication among actors (Corral de Zubielqui et al., 2019). Similar cognition, conventions and shared language can help firms understand ambiguous information (Ferreras-Méndez et al., 2015). Common culture and rules are conducive to grasping the usefulness of knowledge (Inkpen and Tsang, 2005). Therefore, an increase in the level of cognitive capital is expected to improve the quality of the knowledge transferred by the firm.

Therefore, we propose the following:

H2a: Structural social capital facilitates knowledge quality.

H2b: Relational social capital facilitates knowledge quality.

H2c: Cognitive social capital facilitates knowledge quality.

Social capital provides more channels and opportunities for high-quality knowledge transfer among actors. Bari et al. (2019) have pointed out that effective communication and cooperation can enhance the organization’s knowledge reserves and technical skills, thus stimulating creativity. According to the KBV, the high-quality knowledge acquired through effective transfer further accelerates organizations’ knowledge creation and induces better innovation performance (Zhang and Min, 2021). Knowledge quality may play a mediating role in the relationship between social capital and product innovation performance. The KBV emphasizes that the knowledge process accounts for the majority of the innovation process (Grant, 1996a). Improving knowledge quality means acquiring more valuable knowledge to produce new products while bringing them to the market faster, reducing costs and increasing sales in the process (Durmuşoğlu, 2013). It can be said that the quality of knowledge determines the basic quality of product innovation thought. Moreover, the quality of the knowledge transferred between firms affects their dynamic capabilities of new product development (Prange et al., 2018). Based on the above argument, we propose the following hypotheses:

H3a: Knowledge quality mediates the relationship between structural social capital and product innovation performance.

H3b: Knowledge quality mediates the relationship between relational social capital and product innovation performance.

H3c: Knowledge quality mediates the relationship between cognitive social capital and product innovation performance.

**FIGURE 1:**
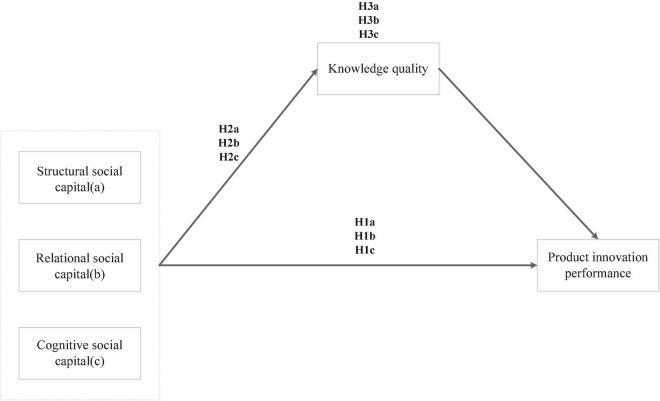
Research model.

Following this logic, we predict the following (please see Figure 1).

## Methodology

### Sample and data collection

Empirical researchers are increasingly concerned that whether the methodology can be replicated. Improving the replicability (e.g., credibility and robustness) would greatly increase the academic value of this study. Brendel et al. (2021) proposed that the replication research is observational and context-dependent. Therefore, the environment in which the sample is located, organization factors, and the selection of respondents are all key factors in ensuring the replicability of the study. Drawing on the suggestion of Brendel et al. (2021), this study strictly defined these factors in the methodology design. Such as, the sample firms were restricted to be Chinese KIFs, and the standards for knowledge-intensive firms were defined; respondents who could make timely and accurate evaluations of variables were designated.

According to Starbuck’s (1992) summary, KIFs have five characteristics: (1) extensive use of knowledge; (2) emphasis on esoteric expertise, exceptional expertise must make important contributions; (3) the definition of expertise is broad, and expertise is embedded in many machines and programs; (4) employ people with specialized expertise; (5) knowledge exists in firms’ routines, cultures and professional culture. In China, firms from the IT, biotechnology, new materials, robot manufacturing and electrical engineering industries fit these characteristics. Firms in these industries were selected as units of analysis in this study. We conducted an investigation in Guangdong Province, where the chosen industry is concentrated. The firms in these industries are most suitable for our examination. First, external knowledge transfer is the key process within their knowledge-intensive activities. Second, these industries are developing rapidly, and innovation occurs frequently (Martin and Salomon, 2003), requiring firms to constantly upgrade their knowledge pools. Furthermore, there are close relationships between the firms in these industries, and these relationships form an inter-organizational network facilitating exchanges of resources and knowledge (Fountain, 1998).

This study selected executives of knowledge-intensive firms from the MBA programs of two universities in Guangdong Province as latent informants. MBA students who held senior positions were solicited to participate in our survey, and 207 of them expressed interest in this research. We required these individuals to confirm that their firms meet our three criteria: (1) they were in an inter-organizational network of knowledge-intensive industries; (2) they were innovation-intensive firms; and (3) they maintained business relations with other firms. Finally, we selected 166 firms and asked one senior executive and one core member of R&D department of each firm to be our respondents. Before collecting the data, we invited 15 senior executives and 3 university scholars in related fields to comment on the clarity and relevance of our questionnaire items to ensure that all the items were representative and well understood.

In the data collection stage, we conducted an online questionnaire survey and sent network links to the questionnaire to all the respondents. A cross-sectional design was adopted in this study. The main study variables of the model were measured with self-reported items. Common method variance (CMV) may arise when a single respondent answers questions corresponding to both independent and dependent variables in a cross-sectional survey with a single measurement background (Podsakoff et al., 2003). Multiple data sources not only reduce the potential threat of CMV but also improve the reliability and validity of investigations by obtaining information from individuals with the most information on the focal subject (Park and Lee, 2014). As mentioned above, the analysis unit of this study is a firm, and firm-level data are collected from the same pair of individuals in each firm: one senior executive and one core member of the R&D department. The senior executives were asked to evaluate three dimensions of social capital (independent variable) and firm age, size and ownership structure (control variable). The core R&D department members were asked to evaluate knowledge quality (mediating variable) and product innovation performance (dependent variable). To better clarify the firm to which each individual belonged, we coded the abovementioned 166 firms. After completing the questionnaire, all the respondents were required to submit their firm codes. The firms that met the criteria for further analysis were required to have at least one questionnaire from a senior executive and one questionnaire from a core R&D department member. Finally, we obtained 159 matching samples, namely, 159 firm senior executives and 159 core R&D department members. The profiles of the individuals and firms are listed in Table 1.

**TABLE 1:** The profiles of respondents and firms.

Item	Frequency	Percent
**The information of informants**		
**Gender**		
Male	237	74.5%
Female	81	25.5%
**Age**		
≤30	20	6.3%
31–35 years	101	31.8%
36–40 years	93	29.2%
41–45 years	68	21.4%
46 years or more	36	11.3%
**The information of firms**		
**Industrial type**		
IT	73	45.9%
New materials	45	28.3%
Biological medicine	28	17.6%
Others	13	8.2%
**Size**		
300 or fewer employees	71	44.7%
300–2,000 employees	74	46.5%
2,000 or more employees	14	8.8%
**Age**		
Less than 5 years 6–20 years 20 years or more	36 93 30	22.6% 58.5% 18.9%
**Ownership**		
State-owned Other	38 121	23.9% 76.1%

### Selection of variables and scales

The variables and scales used in this study were selected on the basis of a literature review (see Table A1 for a list of the items). The responses were rated on a seven-point Likert-type scale ranging from 1 (strongly disagree) to 7 (strongly agree).

#### Social capital

Social capital scale includes structural, relational and cognitive social capital. A four-item scale developed by Molina-Morales and Martínez-Fernández (2010) was used to measure structural social capital. A two-item scale adopted from Ortiz et al. (2018) was employed to measure the level of relational social capital. A four-item scale developed by Ortiz et al. (2018) was used to measure the cognitive social capital. The sample items for structural, relational, and cognitive social capital were “People from your company spend a considerable amount of time on social occasions with people from other firms,” “Has external relationships based on cooperation and mutual trust,” and “Shares goals and projects interests with its external relationships.”

#### Knowledge quality

We adopted four items created by Corral de Zubielqui et al. (2019) to measure knowledge quality. Participants were requested to assess the quality of knowledge transferred between their firm and other external organizations (i.e., timeliness, accuracy, completeness, and adequateness). One sample item was “Knowledge transfers with partners are timely.”

#### Product innovation performance

We used the efficacy and efficiency of product innovation to evaluate the firm’s product innovation performance. Based on the scale developed by Alegre and Chiva (2008), seven items were used to evaluate the product innovation efficacy, and another four items were used to evaluate the product innovation efficiency. Two sample items for efficacy and efficiency were “Replacement of products being phased out” and “Average innovation project development time,” respectively.

#### Control variables

According to previous studies, firm size, age and ownership are important predictors of product innovation because these factors induce differences in resource allocation, managerial competences and knowledge transfer, thus affecting organizational performance (Chen et al., 2014). We measured firm size as the number of employees in each firm (natural logarithm). Firm age was the number of years since a firm was founded. Firm ownership was assessed with a dummy variable (i.e., 1 denoted state-owned firm and 0 denoted private-owned firm).

## Results and analysis

We examined the hypotheses described in our conceptual model (Figure 1) using partial least squares-structural equation modeling (PLS-SEM). The sample size of our survey was small (*n* = 159), and PLS-SEM can be used to overcome shortcomings resulting from a small sample size and skewed distribution (Reinartz et al., 2009). In addition, the relationships between the studied variables are complex (e.g., mediation, moderation, and moderated mediation), and PLS-SEM can provide robust solutions for complex research models (Leal-Rodríguez et al., 2014). Therefore, PLS-SEM may improve our work. SmartPLS 3.0 software was used to examine the hypotheses depicted in the research model (Hair et al., 2013). Then, the outer model was used to evaluate the reliability and validity of the instruments, and the inner model was used to test the hypotheses proposed in the research model.

### Measurement model

We used construct reliability, convergent validity and discriminant validation to estimate the measurement model. The factor loading scores, Cronbach’s alpha, composite reliability (CR) and average variance extracted (AVE) of each item are shown in Table 2. The loading of each factor in the model is greater than 0.7, which exceeds the minimum value of 0.5 suggested by Joseph F. Hair et al. (2013). The Cronbach’s alpha and composite reliability (CR) score of each construct were greater than Bagozzi and Yi (1988) recommended minimum value of 0.7. Moreover, the lowest AVE was 0.701, which is greater than the 0.5 threshold. Based on the above facts, the model had good construct and convergent validity. Table 3 provides the AVE square root and cross-correlation of each construct. We found that the AVE values were significantly greater than the corresponding cross-correlations. Thus, according to the Fornell-Larcker criterion, there was sufficient discriminant validity among the scales.

**TABLE 2:** The indices for construct reliability and convergent validity.

Construct/item	Factor loading	Cronbach’s alpha	AVE	CR
**Structural social capital (SSC)** Molina-Morales and Martínez-Fernández (2010)		0.880	0.735	0.917
SSC1: People from your company spend a considerable amount of time on social occasions with people from other firms	0.860			
SSC2: People from your company spend a considerable amount of time on social events organized by the local community	0.875			
SSC3: A local origin and common academic background of the employees at local firms allow social interactions to take place	0.815			
SSC4: There is an informal network among customers, suppliers and competitors	0.878			
**Relational social capital (RSC)** Ortiz et al. (2018)		0.730	0.788	0.882
RSC1: Has external relationships based on cooperation and mutual trust	0.879			
RSC2: Has external relationships based on cooperation and mutual trust	0.896			
**Cognitive social capital (CSC)** Ortiz et al. (2018)		0.901	0.771	0.931
CSC1: Shares goals and projects interests with its external relationships	0.880			
CSC2: Shares language and a common vision regarding the functioning and factors of success of the environment with external agents (relationships)	0.796			
CSC3: Understands work techniques in a similar way to the external agents with whom it has relationships	0.926			
CSC4: Shares a common culture with external agents from repeated interactions	0.909			
**Knowledge quality (KQ)** Corral de Zubielqui et al. (2019)		0.892	0.755	0.925
KQ1: Knowledge transfers with partners are timely	0.870			
KQ2: Knowledge transfers with partners are accurate	0.851			
KQ3: Knowledge transfers with partners are complete	0.900			
KQ4: Knowledge transfers with partners are adequate	0.863			
**Product innovation performance (PIP)** Alegre and Chiva (2008)		0.957	0.701	0.963
Product innovation efficacy				
PIP1: Replacement of products being phased out	0.912			
PIP2: Extension of product range within main product field through new products	0.795			
PIP3: Extension of product range outside main product field	0.777			
PIP4: Development of environment-friendly products	0.858			
PIP5: Market share evolution	0.825			
PIP6: Opening of new markets abroad	0.807			
PIP7: Opening of new domestic target groups	0.833			
Product innovation efficiency				
PIP8: Average innovation project development time	0.848			
PIP9: Average number of working hours on innovation projects	0.852			
PIP10: Average cost per innovation project	0.838			
PIP11: Global degree of satisfaction with innovation project efficiency	0.847			

**TABLE 3:** The indices for discriminant validation.

Constructs	SSC	RSC	CSC	KQ	PIP
Structural social capital (SSC)	**0.858**				
Relational social capital (RSC)	0.466	**0.888**			
Cognitive social capital (CSC)	0.343	0.709	**0.878**		
Knowledge quality (KQ)	0.342	0.534	0.559	**0.869**	
Product innovation performance (PIP)	0.486	0.643	0.603	0.613	**0.837**

Bold values show the square root of AVE for the corresponding construct.

### Structural model

**FIGURE 2:**
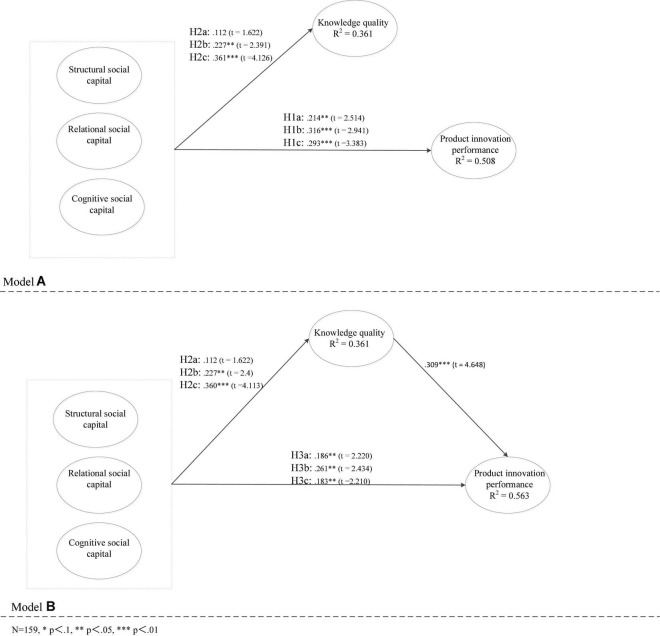
Models used to test mediation.

We used the procedure recommended by Hartono et al. (2010) to verify the mediating effect shown in Figure 2. There were two criteria for full or partial mediation: (1) social capital had a significant effect on product innovation performance and knowledge quality (Model A, baseline model), (2) knowledge quality had a significant effect on firm product innovation performance, and the effect of social capital on firm product innovation performance was significantly reduced (partial mediation) or no longer significant (full mediation) (Model B, mediation model).

Each path coefficient was calculated via a bootstrapping method with 5,000 steps. Figure 2 depicts the correlation coefficients between the variables in each model. Structural social capital had a positive effect on innovation performance (*r* = 0.214, *t* = 2.514, *p* < 0.05), relational social capital had a significant positive effect on product innovation performance (*r* = 0.316, *t* = 2.941, *p* < 0.01), and cognitive social capital had a significant positive effect on product innovation performance (*r* = 0.293, *t* = 3.383, *p* < 0.01), supporting H1a, H1b and H1c. Moreover, the coefficients of the paths from relational social capital to knowledge quality (*r* = 0.227, *t* = 2.391, *p* < 0.05) and cognitive social capital to knowledge quality (*r* = 0.361, *t* = 4.126, *p* < 0.01) were significant, supporting H2b and H2c. In Model 2, knowledge quality had a positive effect on product innovation performance (*r* = 0.309, *t* = 4.648, *p* < 0.01). Additionally, the coefficients of the paths from relational social capital to product innovation performance (*r* = 0.261, *t* = 2.434, *p* < 0.05) and cognitive social capital to product innovation performance were significantly reduced (*r* = 0.183, *t* = 2.210, *p* < 0.05). The criteria for partial mediation were met, supporting H3b and H3c.

However, the results did not support H2a and H3a. The relationship between structural social capital and knowledge quality was not significant (*r* = 0.112, *t* = 1.622, *p* > 0.1). Some traits of structural social capital may play a role here. On the one hand, with the enhancement of structural social capital, social network owned by a firm can be expanded and further increases the possibility of acquiring essential external information that are critical for improving knowledge quality. On the other hand, a larger network makes knowledge base of a firm become more diverse and complex (Demirkan et al., 2013). A firm needs to invest more specific resources and develops more specialized capabilities to identify complex and fragmented knowledge (Woolcock and Narayan, 2000; Kim and Shim, 2018). From the perspective of controlling management costs, if a firm with limited resources spends too much time and money identifying the fragmented external knowledge, this firm can hardly balance other key knowledge activities (e.g., integrating diverse knowledge) that facilitate knowledge quality (Demirkan et al., 2013; Zahra et al., 2020). Therefore, resources stress resulting from structural social capital may reduce a firm’s ability that improves knowledge quality. Based on this logic, it can be explained that the impact of structural social capital on knowledge quality is not significant.

## Discussion

Although previous studies have provided empirical evidence for the effect of social capital on innovation through knowledge transfer, little attention has been given to the quality of transferred knowledge, and this issue has not been studied in the context of Chinese knowledge-intensive industries. The main contribution of this study is that it offers a detailed picture of the links between different dimensions of social capital, knowledge quality and product innovation performance in Chinese knowledge-intensive firms.

### Theoretical implications

This paper contributes to the innovation and knowledge management literature in three ways. First, this study extends existing research on firm innovation by investigating the effects of three sub-constructs of social capital (i.e., structural, relational and cognitive social capital) on product innovation performance. Previous studies on the social capital-innovation performance relationship have operationalized social capital as a single construct (Carmona-Lavado et al., 2010; Duodu and Rowlinson, 2020), which can hardly distinguish the separate influences of three dimensions of social capital. This study provides empirical evidence on the importance of structural, relational and cognitive social capital in predicting firm innovation.

Second, we study the relationship between social capital and the quality of transferred knowledge, adding support to the literature on knowledge transfer management of firms. Social capital is well known as an important influencing factor of knowledge transfer. However, most studies only focused on the quantity of transferred knowledge (i.e., knowledge sharing) without considering knowledge quality (another critical dimension of knowledge transfer) (Filieri et al., 2014). The current study examines the effects of three dimensions of social capital on knowledge quality. The results show that both relational social capital and cognitive social capital have a positive impact on transferred knowledge quality, providing a more thorough understanding about the role of social capital in shaping the knowledge transfer process of the firm.

Third, our research contributes to the KBV literature by examining the mediating effect of knowledge quality in the relationship between social capital and firm innovation. The findings show that both relational social capital and cognitive social capital can impose a positive indirect effect on product innovation performance through knowledge quality. This result further expands KBV theory by demonstrating that knowledge quality can serve as another knowledge-based resource that explains the internal mechanism of how social capital facilitates innovation of firms.

### Practical implications

Our findings also have practical implications for firm managers in Chinese knowledge-intensive industries. Managers should understand that good inter-organizational social capital management allows firms to develop dynamic capabilities related to high-quality knowledge transfer. The ultimate goal of this process is to efficiently use resources, improve innovation performance and respond to changes in the external environment. In addition, managers need to consider the different impact mechanisms of various dimensions of social capital on innovation performance.

The quality of transferred knowledge partially mediates the positive effects of relational social capital and cognitive social capital on product innovation performance. Firm managers should emphasize knowledge management with external stakeholders. Managers should consider not only the quantity of knowledge obtained from external relations but also the quality of such knowledge to gain more valuable resources.

### Limitations and future research directions

As with other studies, we can point out the following limitations of this study. First, all the findings need to be interpreted within the limitations of this exploratory study. In particular, although our sample size (*n* = 159) meets the minimum sample size requirements suggested by Barclay et al. (1995), is still less than the average sample size (*n* = 211) of studies using Smart-PLS (Hair et al., 2012). Scholars should use larger samples to test the stability of our findings. In addition, the sample is limited to Chinese knowledge-intensive firms; thus, the findings may not apply to firms in different industries and different countries.

## Conclusion

This paper addresses the issue of whether and how social capital influences the product innovation performance of Chinese knowledge-intensive firms. Using data collected from such firms, we study the relationship between social capital and product innovation performance and the role of knowledge quality in this relationship. Our findings confirm that social capital has a positive effect on product innovation performance. The three dimensions (structural, relational and cognitive dimensions) of social capital have positive impacts on product innovation performance. Both the relational dimension and the cognitive dimension have positive indirect effects on product innovation performance through knowledge quality, whereas the mediating role of knowledge quality in the relationship between the structural dimension and product innovation performance is not significant. These results enrich our understanding of the different mechanisms underlying the effects of different social capital dimensions on product innovation performance. Our research not only helps expand the relevant literature on social capital and knowledge management but also provides guidance for the product innovation of Chinese knowledge-intensive firms.

## Data availability statement

The raw data supporting the conclusions of this article will be made available by the authors, without undue reservation.

## Author contributions

XZ contributed to the conceptualization, investigation, formal analysis, and writing – original draft. MM contributed to the writing – original draft, methodology, software, and investigation. ZZ contributed to the investigation and supervision. All authors contributed to the article and approved the submitted version.

## Appendix

**TABLE A1:** Research items.

Construct	Item (from 1-strongly disagree to 7-strongly agree)
**Structural social capital (SSC)**	
	People from your company spend a considerable amount of time on social occasions with people from other firms
	People from your company spend a considerable amount of time on social events organized by the local community
	A local origin and common academic background of the employees at local firms allow social interactions to take place
	There is an informal network among customers, suppliers and competitors
**Relational social capital (RSC)**	
	Has external relationships based on cooperation and mutual trust
	Has external relationships based on cooperation and mutual trust
**Cognitive social capital (CSC)**	
	Shares goals and projects interests with its external relationships
	Shares language and a common vision regarding the functioning and factors of success of the environment with external agents (relationships)
	Understands work techniques in a similar way to the external agents with whom it has relationships
	Shares a common culture with external agents from repeated interactions
**Knowledge quality (KQ)**	
	Knowledge transfers with partners are timely
	Knowledge transfers with partners are accurate
	Knowledge transfers with partners are complete
	Knowledge transfers with partners are adequate
**Product innovation performance (PIP)**	
Product innovation efficacy	
	Replacement of products being phased out
	Extension of product range within main product field through new products
	Extension of product range outside main product field
	Development of environment-friendly products
	Market share evolution
	Opening of new markets abroad
	Opening of new domestic target groups
Product innovation efficiency	
	Average innovation project development time
	Average number of working hours on innovation projects
	Average cost per innovation project
	Global degree of satisfaction with innovation project efficiency
